# Providing “Free” Access to Dialysis and Transplant to the Disfranchised. A Sustainable Model for Low and Low Middle Income Countries (LMICs)

**DOI:** 10.3389/ti.2023.11290

**Published:** 2023-07-11

**Authors:** Mirza Naqi Zafar, Syed Adibul Hasan Rizvi

**Affiliations:** ^1^ Department of Pathology, Sindh Institute of Urology and Transplantation, Karachi, Pakistan; ^2^ Department of Urology, Sindh Institute of Urology and Transplantation, Karachi, Pakistan

**Keywords:** free model, dialysis, transplantation, disfranchised, LMIC

## Abstract

Pakistan is a low-middle income country where incidence of End Stage Kidney Disease (ESKD) is 100–150 per million population (pmp). Paucity and high costs of renal replacement therapy (RRT) renders the majority disfranchised, since the dialysis rate is 15 pmp and the transplant rate is 4–5 pmp. In view of this, our center started an integrated dialysis and transplant program where all treatment is provided “Free of Cost” to all patients, with lifelong follow-up and medications. The model is based on the concept of community-government partnership funded by both partners. The annual contribution in 2021 was $37.4 million. >1,500 patients were dialyzed daily, and 6–8 received transplants weekly. Of the 6,553 transplants performed between 1985–2021, 988 (15%) were children. Overall, the 1 and 5-year graft survival rate was 97% and 88%. The donor clinic has 3,786 donors in regular yearly follow-up for up to 30–35 years where ESKD prevalence is 0.29%. Access to dialysis was increased by establishing six satellite centers reducing patient time and travel costs. Cost reductions by dialyzer reuse and generic drugs resulted in an annual saving of $5.8 m. This sustainable model has overcome the inherent socio-economic, logistic, cultural, and gender biases in RRT in LMICs. It has provided RRT with equity to the disfranchised in Pakistan and can be replicated in other LMICs with community-government support.

## Graphical Abstract

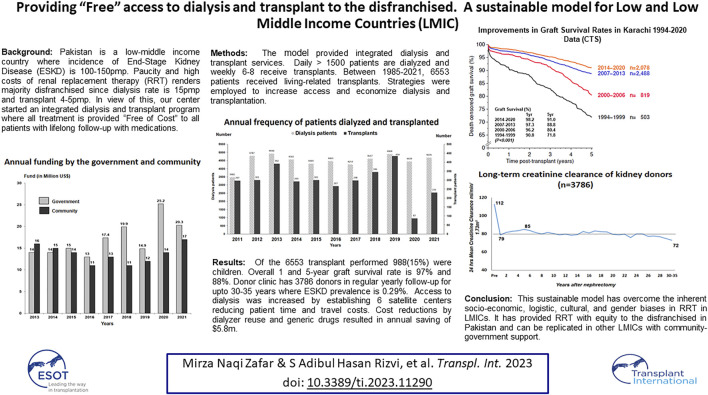

## Introduction

Renal replacement therapy (RRT) through dialysis or transplantation are the standard of care life-saving therapies for patients with End Stage Kidney Disease (ESKD). In a report by Global Kidney Health Atlas of 160 countries, the incidence of ESKD in High Income Countries (HIC) was 149 per million population (pmp) as compared to 129 pmp in low income countries (LIC) [1]. The average rate of RRT globally was 759 pmp. The rate in HIC was 969 pmp, LMIC 321 pmp, and LIC 4.4 pmp [1]. In LMICs in the neighboring region of South Asia, including India, Pakistan, Bangladesh, and Sri Lanka, the ESKD incidence is 100–160 pmp, the RRT rate is 20–70 pmp, and the transplantation rate is 1–10 pmp [2].

There is therefore a disparity between the incidence of ESKD and RRT in LMICs. Firstly, due to economic constraints where the government expenditure on health is <1%–4.0% of the gross domestic product (GDP). Secondly, *per capita* income ranges from $3,000–24,000 per year and 10%–50% of people live below the poverty line on <$2/day [2]. Thirdly, 25%–65% of the population live in rural settings and have problems accessing dialysis and transplant centers situated in cities [2, 3]. Finally, if they are able to access treatment centers, only 20%–30% get free RRT in LMICs. The rest have to pay, and costs are often beyond their reach as Haemodialysis costs $13,510/year/person, kidney transplant (1st year) costs $11,746, and kidney transplant (after 1st year) costs $5,659/year/person [1].

Pakistan is an LMIC with a population of 221 million where GDP *per capita* is $1,658/year. The government expenditure on health is 1.2% of GDP, 50% live below the poverty line on < $2 a day, and 65% of the people live in rural settings [4]. Estimated ESKD incidence is 100–150 pmp and in terms of RRT, the dialysis rate is 15 pmp, and the transplant rate is 4.5 pmp [2, 5]. The cost of hemodialysis for 1 year is $4,873, where 51%–75% are out-of-pocket expenses [6]. The cost of a transplant is ∼$10,000 in the private sector [2].

In this backdrop of disparity between the incidence of ESKD and RRT therapy, a model based on Community Government Partnership was established at our center to provide an integrated dialysis and transplant service “free of cost” to the disfranchised of the country irrespective of caste, color, creed, religion, and socioeconomic status [7]. The guiding principles of the model are equity, transparency, accountability to its supporting partners, and to provide the best care to all its patients with life-long follow-up with medications [8].

In this paper, we describe the achievements of our model and strategies for sustainability. Its ability to provide equitable RRT by overcoming problems of economics, accessibility, gender, and cultural bias by “free of cost care.”

## Patients and Methods

### Model of Community Government Partnership

The Institute is a public sector organization where the government provided land, infrastructure, equipment, utilities, and staff salaries. The community was mobilized to support the services offered in kind or cash. A trust was established in the 1980s where notables of society, professionals, and government officers formed a Board of Governors. The government in view of free services upgraded a Urology Ward to an Institute of Urology and Transplantation by an act of the provincial parliament in 1991. The Institute receives a yearly grant-in-aid from provincial budget and another source of funds is contribution from the community. It runs as an autonomous body accountable to the community and government. The accounts are audited by independent firms of auditors and presented to both the partners of the model.

### Dialysis

The institute has 350 dialysis machines, of which 25 are dedicated to Hepatitis B patients. In the year 2021, there were 4,676 registered patients who were dialyzed 2–3 times a week using bicarbonate solution. There are eight dialysis centers (two in the main campus and six satellite centers) working 6 days a week. Emergency dialysis is available 24/7. Dialysis is performed by lines initially and arterio-venous fistula (AVF) are made within the first 3 weeks for maintenance dialysis.

Adequacy of dialysis is checked clinically and by urea reduction rate (URR) [(Pre dialysis urea—Post dialysis urea) × 100/Pre dialysis urea] to be in the range 65%–70%. During dialysis, venous pressure is checked to be maintained at half of the flow rate of 250 mL/h. Routinely urea and creatinine are checked every 4 weeks.

#### Cost Saving Strategies in Dialysis

Dialyzer reuse was introduced in 1996, except for Hepatitis B-positive patients. Dialyzer reuse is stopped when the reprocessing machine gives a Bundle Pressure of <80% or reports a pressure failure. Basic dialysis machines are used without profiling or dialysate modeling. Dialysis fluid is prepared in-house from imported reagents in a dedicated department with strict quality control by daily monitoring of constituents and cultures.

### Transplantation

#### Recipient Follow-Up

A total of 6,553 renal transplants were performed between 1985 and 2021 by live related donors. Since 1994, 5883 transplants were reported to the Collaborative Transplant Study (CTS), a transplant outcome registry, Heidelberg University [9]. A total of six to eight transplants are performed weekly. All recipients are followed-up on in a dedicated clinic with a volume of 80–120 patients per day. The clinic comprises surgeons, nephrologists, specialists in Internal medicine, dieticians, and medical social workers. Laboratory facility, ultrasound, and pharmacy are part of the clinic. Patients are given immunosuppression medication at each visit for 1–3 months depending on their place of residence in the country.

#### Immunosuppression

The protocol evolves as and when the drugs become available in the country. A detailed protocol has been published before [10]. Immunosuppression protocol is based on HLA match. Briefly, all patients with a 3–6 antigen match receive a triple-drug regimen comprising Cyclosporine (CyA)/Azathioprine (AZA) and Prednisolone. CyA is given at 6 mg/kg body weight while paediatric patients receive 8 mg/kg. Target blood levels for CyA are 200–250 ng/mL. Dose reduction by 3 mg/kg is undertaken in patients who are rejection free at 3 months with a target level of 150–200 ng/mL and 2-h level of 800–1,000 ng/mL.

Recipients with poor match (0–2 antigen) and panel reactive antibodies (PRA >30% are given Tacrolimus (TAC), Prednisolone, and Mycophenolate Mofetil (MMF) as initial therapy along with induction with Antithymocyte Globulin (ATG). TAC is given at 0.15 mg/kg with a target level for the first 1–3 months of 8–10 ng/mL. Dose reduction to 0.1 mg/kg is considered at 3 months if the patient is rejection free. Interleukin 2 Antagonist (IL-2) is given to all children <12 years of age. Biopsy-proven graft rejections are treated with methylprednisolone boluses. Methylprednisolone resistant acute rejections are treated by ATG 3–5 mg/kg for 10–14 days. All graft dysfunction are evaluated by drug levels, Color Doppler, and graft biopsy. Patients are monitored for urinary tract infections (UTI), cytomegalovirus (CMV), tuberculosis, hepatitis B and C, and other infections when indicated.

#### Cost Saving Strategies in Immunosuppression

Patent drugs were replaced by generics as and when they became available in Pakistan. CyA was replaced in 1999 and generic Tacrolimus was introduced in 2002. Bioequivalence studies were undertaken by one-to-one conversion for CyA and TAC and area under the curve (AUC) for CyA with a 6-point sampling.

#### Tissue Typing

Initially, tissue typing was done on microlymphocytotoxicity assay on 60 well Human Leucocyte Antigen (HLA) Class I and Class II Terasaki Plates. Thereafter since 1994, 120 Sera Plates were used for Class I and Class II purchased from CTS Heidelberg Germany. In 1996 CTS sequence-specific primers were used for Class II typing. Antibody screening is by microlymphocytotoxicity assay and flow cross-match for T and B cells was added in 1994. PRA were initially tested on 60 well cell plates and in 2010, Luminex platform was added for pre and post-transplant antibody screening for HLA Class I, and Class II.

#### Donor Selection

Donors are selected according to the guidelines of the Amsterdam Forum and according to the protocol published before [11, 12]. Donors are genetically related or spouses aged between 18–60 years in most cases. All the eligible donors are seen in the pre-transplant donor clinic by physicians, nephrologists, surgeons, medical social workers, and psychologists. They are counseled according to their socio-cultural, educational background, vocation, and family structure. Prospective donors are made to interact with kidney donors to address their apprehensions about future health issues.

#### Donor Follow-Up

Donors are seen weekly in the first month after donation, thereafter 3 times monthly for a year, and then yearly. Up to 2021, 5,185 donors had registered in the donor clinic for regular follow-up where they are assessed for hypertension, renal function, Lipidemia, liver functions, kidney ultrasound, urine analysis and culture, and 24-h urine for creatinine clearance (CrCl) and protein excretion, and any other medical care as needed. All medications for any condition are provided free to the donors.

### Statistical Analysis

All the data were entered and analyzed in SPSS version. 21.0. Descriptive statistics were used to summarize the normally distributed continuous variables as means and standard deviations and non-normally distributed variables as median (IQR). Categorical variables were reported as count and percentages. Kaplan Meier survival function and analysis was performed for comparison of survival curves, and a log-rank test was used. A *p*-value <0.05 was considered to be statistically significant.

A dollar rate of Rupees 230 to a dollar is used for conversion purposes.

## Results

### Strategies for Sustainability of the Model

**FIGURE 1:**
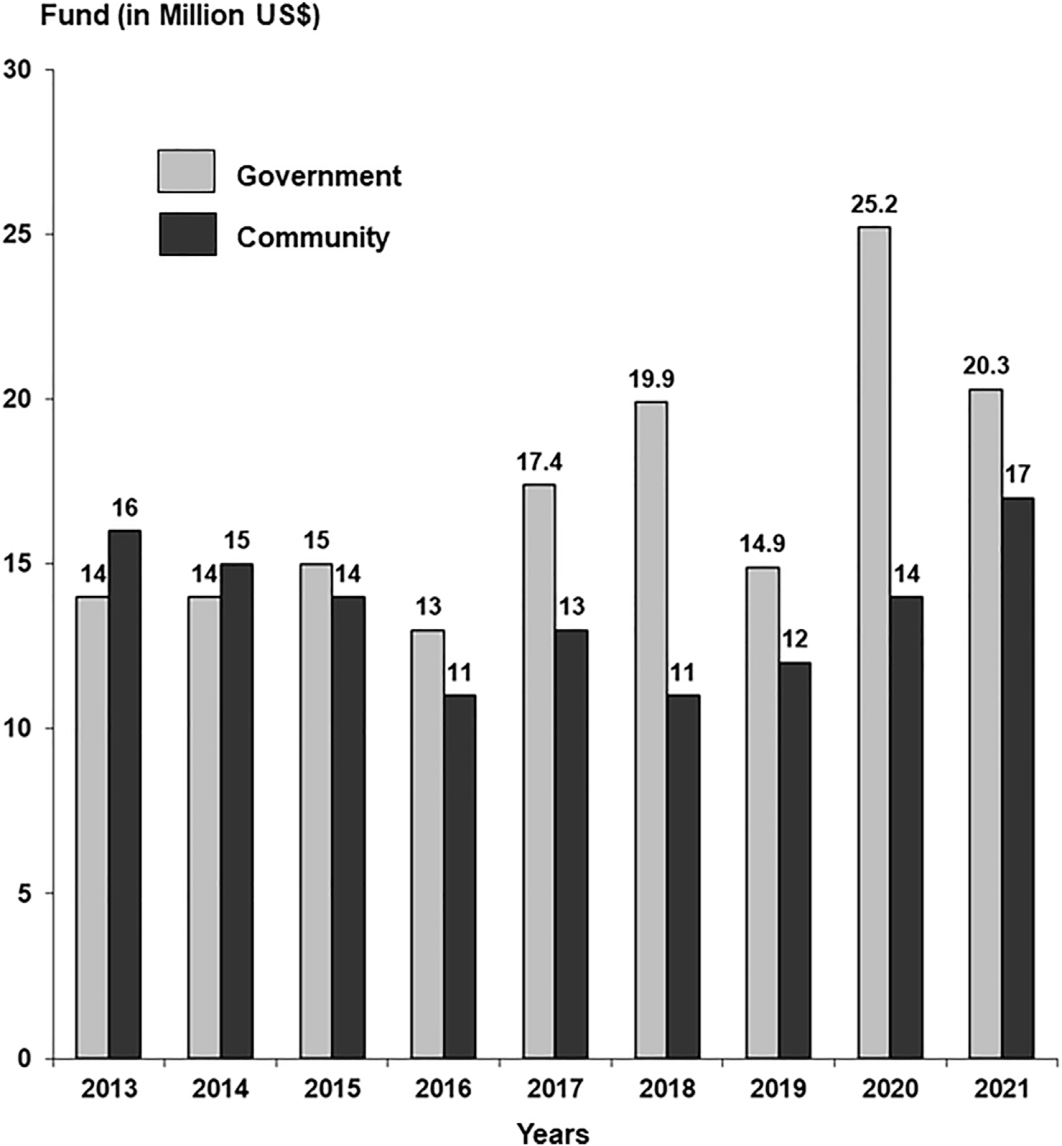
Annual funding by the government and community.

The model of Community-Government Partnership has been operative for more than 4 decades with increasing support from the government and community. The contributions of the two partners for the last 9 years are given in Figure 1. The overall funding in 2021 was $37.3 million. The development of the model was gradual, in which the government provided infrastructure and staff salaries while the community was asked to donate in kindness or cash to run services. The community was engaged through press and electronic media for donations highlighting the free services to the poor, and personally by presentations in social clubs, business houses, corporations, and industries. A number of schemes were introduced to fund costs incurred for the treatment and expansion of the facilities. The government supported by providing tax benefits on donations to community services or foundations. The schemes included 1) patient sponsorship, e.g., dialysis for a year or immunosuppressive drugs for a year 2) Sponsor equipment scheme, e.g., an ECG machine, a laboratory analyzer, an operation table, an X-ray Unit, a dialysis machine, etc. In time, due to the free treatment provided to thousands of patients, the Institute become a focal point for philanthropists, corporations, and business houses.

The third scheme was Sponsor a unit. The scheme resulted in the establishment of a 20-machine dialysis unit for Hepatitis B patients worth US$ 0.7 million in 1999, an Electron microscopy unit worth $ 1 million in 1994, and a lithotripsy unit in 1988 worth $0.8 million. A business house funded construction of a 6-storey building for Dialysis and Transplantation worth $5 million in 1990. In 2000 another business house constructed a 6-storey Oncology center fully equipped with radiation therapy worth $7 million and a 14-storey Transplant Centre fully equipped with four theatres worth $ 15 million in 2016.

The credibility of the Institute being established has helped maintain contributions of the community. The donors are kept informed of the institute activities by a quarterly newsletter that has been running since 1994 with a current distribution of 40,000. In the last decade, social media platforms like Facebook and Twitter disseminate the institute’s awareness programs and services to keep the supporters up to date.

The hallmark of this sustainability is the transparency of services, equity in treatment, and state of art treatment facilities that have made the Institute the largest dialysis and transplant center in the country. All facilities are under one roof and services have expanded to cater for Gastrointestinal Diseases, Hepatology, Cardiology, Internal Medicine, Oncology, Laboratory Medicine, Radiology, and Radiotherapy. Sustainability of the model is shown by the growth of services from 2010 to 2021 in Table 1.

**TABLE 1:** Growth of services at the Institute (2011–2021).

Parameters	2011	2021
No. of patients	770,478	2,960,217
Outpatients	202,456	426,328
Inpatients	33,743	61,034
Emergency	92,102	150,025
Minor and major surgical procedures	66,146	109,863
Dialysis sessions	187,284	410,969
Total Transplants from 1986	3,228	6,271
Radiology tests	203,216	596,533
Laboratory investigations	6,145,004	11,211,665
Medical Costs ($ million)	4.3	10.2
Total staff	1,440	3,012

### Haemodialysis Services

**FIGURE 2:**
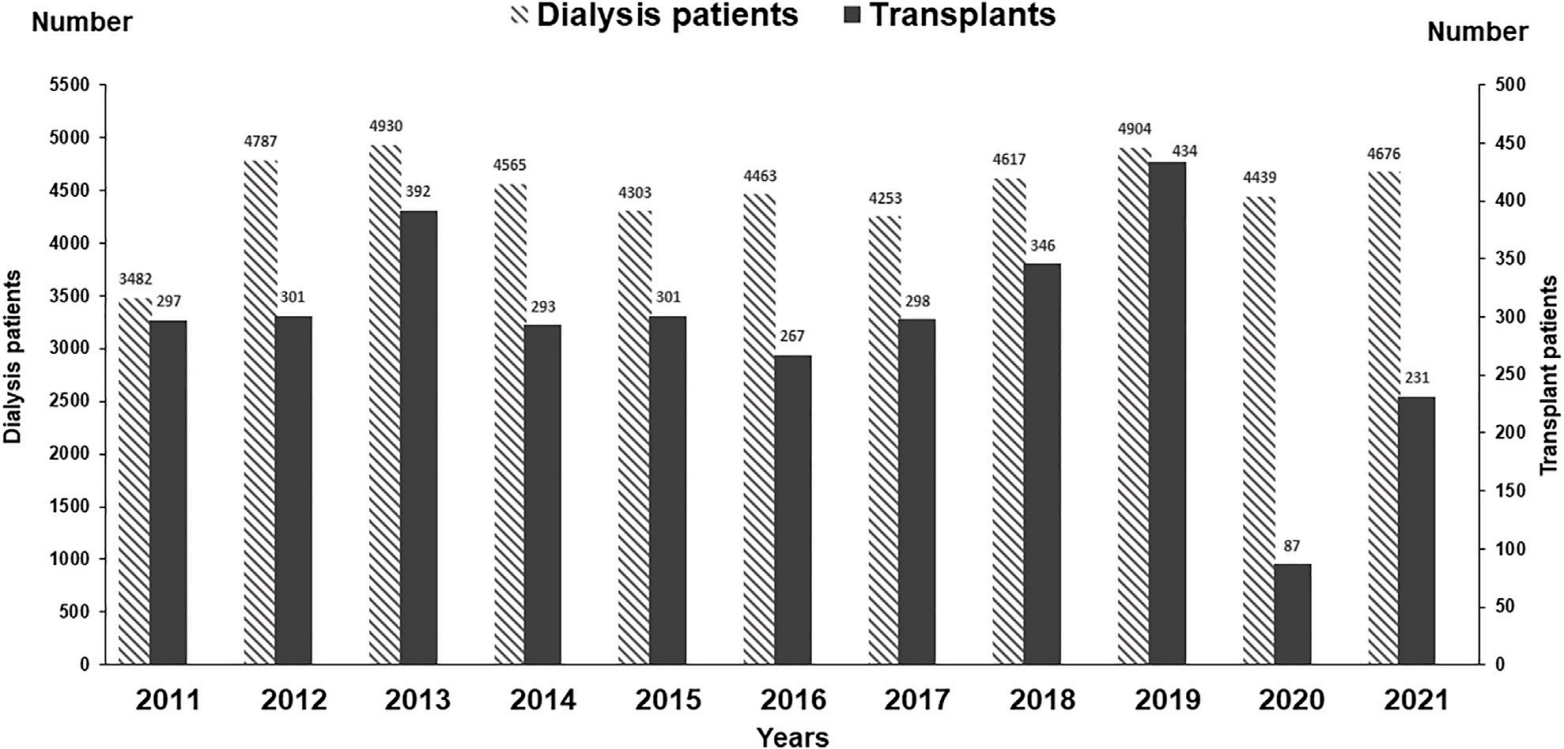
Annual frequency of patients dialyzed and transplanted.

In 2021, a total of 4,676 patients registered for dialysis. Of these, 401 presented with acute kidney injury (AKI), 375 recovered and 26 developed ESKD, 306 presented with advanced stage disease with multi organ failure and died, and 3,969 were on regular dialysis. Of the 3,969 active patients, 401 (10.1%) were children ≤18 years of age with a mean age of 13.0 ± 3.6 years (Range 3–18) and 63% were male. The mean age of adult patients was 44.49 ± 15.0 years (Range 19–89), where 60% were men. The number of patients registered yearly in the last 11 years is given in Figure 2. Overall, 410,969 dialysis sessions were performed in 2021.

### Increasing Accessibility—Dialysis at the Doorstep

Registered patients come from long distances, 16% from 1500 km, 26% from 1000 km, 17% from 500 km, and 30% from within 100 km. Many cannot afford the travel costs of $10–30 from other cities for the 2-3 weekly dialysis. Therefore, many patients live in tents on footpaths near and around the Institute. To cater to these patients and increase accessibility, the institute established 4 satellite dialysis centers in Karachi with a total of 148 machines. Two centers were established in other cities, Sukkur 550 km away with 44 machines and Larkana 450 km away with 26 machines. In 2021, there were 1875 patients on regular dialysis these centers. Satellite centers have resulted in substantial savings in time and travel costs to the patients. Patients residing near and around Sukkur and Larkana reach these centers within 1 h as compared to 7–8 h to Karachi and travel costs were reduced from $10–30 to $1–2 per daily visit.

### Economizing Dialysis

Simple dialysis machines are used which do not have built-in blood pressure, KT/V (K = Urea Clearance of dialyzer (mL/min), T = time in minutes and V = Volume of fluid removed in ml) and disinfection system or endotoxin filter. These machines cost $5,500 as compared to $8,500 for machines with monitors. The dialysis fluid is prepared in-house, where the cost of each dialysis is $1. Dialysis reuse on automatic processors allows a medium reuse of up to 7.0 times. The cost of a dialyzer is $4.5, and reuse reduces the cost to $0.64/dialysis. Considering yearly dialysis sessions in 2021, the cost without reuse would be $1.849 million however with reuse the cost is $0.264 million, a saving of $1.58 million/year.

### Renal Transplantation

A total of 6,553 transplants were performed between 1985–2021 using living related donors. The number of transplants performed in the last 11 years is given in Figure 2. The activity was stopped for 6 months during COVID-19 pandemic in 2020. Of the 6,553 transplants, 988 were pediatric transplants ≤18 years and 601 were spousal transplants. The demographics, clinical characteristics, and outcomes of 6,553 transplants performed between 1985–2021 are given in Table 2. The overall mean age of recipients was 29.15 ± 10.1 years (Range 2–62) with 78% men. The mean age of pediatric transplants was 14.6 ± 3.1 years (range 2–18) whereas 72% were men. The primary disease was unknown in the majority (53.47%) as patients present late with small shrunken kidneys. The mean age of donors was 34.2 ± 9.6 years (Range 18–66) where men were 56%. In the majority (82.65%), initial maintenance immunosuppression was by CyA/AZA/Steroids. Acute rejections were reported in 17%. The main post transplant infections were CMV in 35.8%, recurrent UTIs in 17.6%, and tuberculosis in 14.3%. The majority of the CMV infection 2018 (86%) occurred between 3–6 months post-transplant. Recurrent UTI in the first 6 months post-transplant and TB beyond 1 year transplant. Overall, 1 and 5-year graft survival was 97% and 87%. The main causes of 698 graft losses were Interstitial fibrosis and tubular atrophy (IFTA) in (54%), acute rejection (14%), recurrence of disease (5%), infections (8%), and death with functioning graft (19%). Overall, 1 and 5 years patient survival was 97% and 88%. The main cause of death was infection in 58%.

**TABLE 2:** Demographics, clinical characteristics, and outcomes of renal transplant recipients (*n* = 6,553).

Parameters	Results
Overall Age (years, mean, SD)	29.15 ± 10.19
Adult > 18 (years, mean, SD)	31.72 ± 8.76
Paediatric ≤ 18 (years, mean, SD)	14.66 ± 3.18
Spousal (years, mean, SD)	36.65 ± 7.75
Pediatric up to 18 years (n, %)	988 (15.1)
Spousal (*n*, %)	601 (9.2)
Gender (*n*, %)	
Overall Male (*n*, %)	5,154 (78.7)
Children Male (*n*, %)	712 (72.1)
Spousal Male (*n*, %)	510 (84.9)
Primary renal disease (*n*, %)	
Glomerulopathies	1,361 (20.76)
Congenital/Urologic/Cystic	340 (5.19)
Hypertension	678 (10.35)
Diabetes	102 (1.56)
Stone Disease	568 (8.67)
Unknown	3,504 (53.47)
Time on dialysis (months, median, IQR)	5 (IQR: 3–10)
Donor Age (mean, SD)	34.28 ± 9.67
Male (*n*, %)	3,690 (56.3)
Female (*n*, %)	2,863 (43.7)
Donor Gender	
Paediatric Transplants, Females	571 (57.8)
Spousal Transplants, Females	510 (84.9)
HLA Match (*n*, %)	
4–6	3,771 (57.5)
3	1,988 (30.3)
0–2	794 (12.1)
Panel reactive antibodies (PRA) (*n*, %)	
0%–10%	6,083 (92.8)
>10%	470 (7.2)
Immunosuppression (*n*, %)	
Induction therapy (ATG/IL-2)	1,120 (17)
Initial Maintenance (*n*, %)	
Cyclosporine/Aza/Steroid	5,416 (82.65)
Tacrolimus/MMF/Steroid	668 (10.19)
Cyclosporine/MMF/Steroid	239 (3.6)
Tacrolimus/Aza/Steroid	555 (8.4)
mTOR Inhibitors	586 (8.9)
Acute rejection (*n*, %)	1,141 (17)
Post-Transplant Chronic infections (*n*, %)	
Tuberculosis	937 (14.3)
Recurrent UTI	1,156 (17.6)
HCV	819 (12.5)
CMV	2,346 (35.8)
1 and 5-year Graft Survival (*n*, %)	
Overall	6,553, 97% and 87%
Paediatric	988, 96% and 85%
Spousal	601, 97% and 85%
1 and 5-year Patient Survival (*n*, %)	
Overall	6,553, 97% and 88%
Paediatric	988, 97% and 90%
Spousal	601, 98% and 88%

#### Economizing Immunosuppression

Firstly, generic drugs are used instead of patent drugs to reduce costs and secondly, immunosuppression protocol is based on HLA match where 82.6% recipients are given CyA/AZA/Steroids and 18% TAC/MMF or AZA/Steroids. Induction by ATG or IL-2 is given to 17% of the patients. The cost of CyA/AZA/Steroids for the first year is $650 while for TAC/MMF/Steroids the cost is $1,300. The total saving using CyA/AZA is around $4.2 million.

**FIGURE 3:**
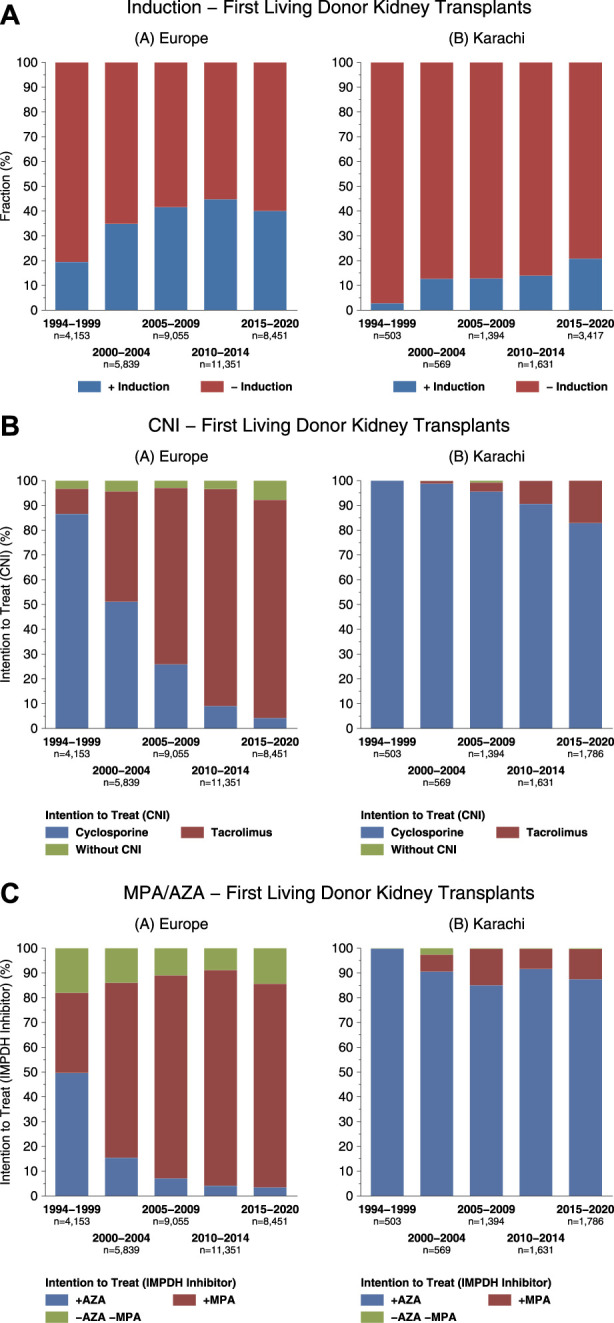
Comparison of immunosuppression between Europe and Karachi - First living donor kidney transplants **(A)**: Induction Therapy **(B)**: Calcineurin Inhibitors **(C)**: IMPDH Inhibitors.

**FIGURE 4:**
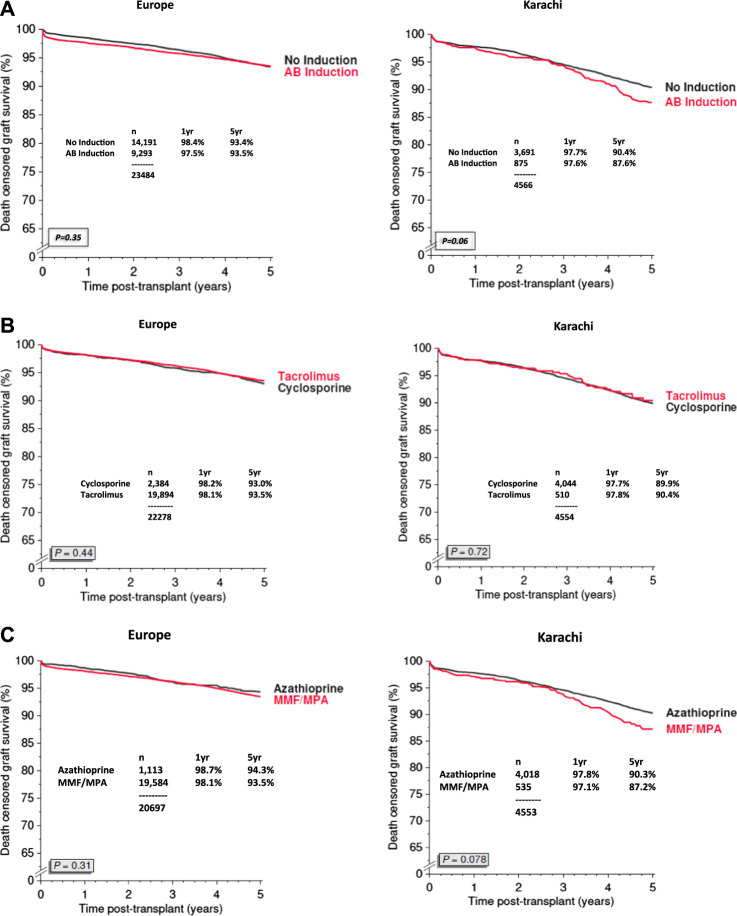
Graft survival based on Immunosuppression regime between Europe and Pakistan (2007–2020) **(A)**: Induction Therapy **(B)**: Calcineurin Inhibitors **(C)**: IMPDH Inhibitors.

**FIGURE 5:**
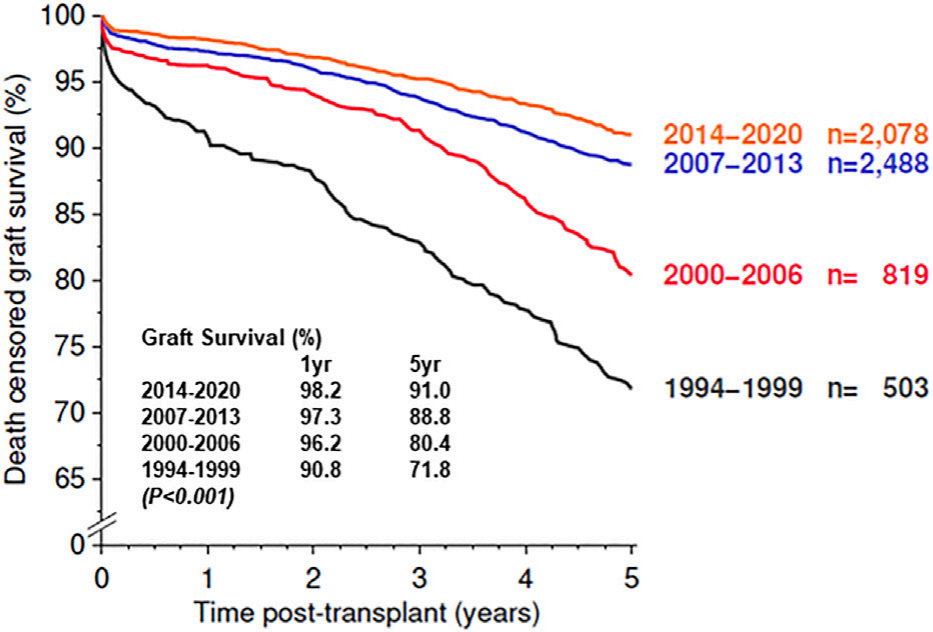
Graft survival of First Living Donor Kidney Transplant in Karachi.

**FIGURE 6:**
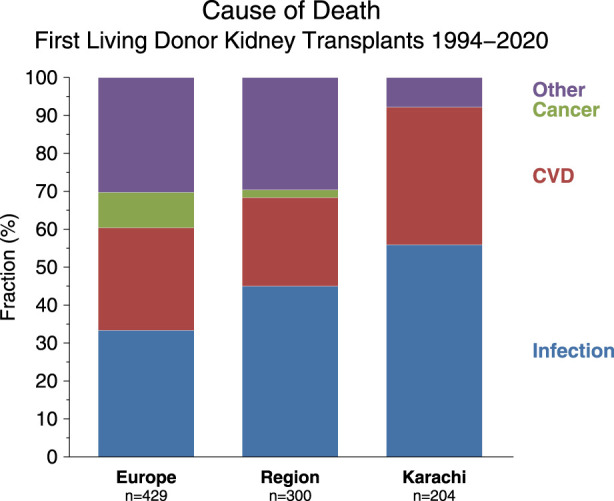
Comparison of cause of death between Europe, Middle East (“Region”), and institute (“Karachi”).

We have compared the immunosuppressive drugs used in our Institute with those in Europe and their impact on graft outcomes. Figure 3 shows the use of different Calcineurin and Inosine-5′-monophosphate dehydrogenase (IMPDH) inhibitors for first living donor transplants between Europe and our Institute from 1994–2020 (Courtesy CTS) [9]. The comparison is based on 5,883 transplants at our Institute and 38,949 in Europe. Induction therapy by ATG or IL-2 in Karachi is used in 19% vs. 40% in Europe (Figure 3A). Calcineurin Inhibitor CyA is used in 88% in Karachi while TAC is used in 89% in Europe (Figure 3B). IMPDH inhibitor AZA is used in 88% in Karachi while MMF is used in 94% in Europe (Figure 3C). A comparison of death-censored graft survival with different Immunosuppressive drugs is in Figure 4. Graft survival rates are similar in induction vs. no induction both in Europe and Karachi (Figure 4A). TAC vs. CyA (Figure 4B) and AZA vs. MMF/MPA (Figure 4C). Using cheaper immunosuppressive drugs and HLA driven immunosuppression we are able to achieve similar graft survival rates between Induction vs. No Induction, CyA vs. TAC, and AZA vs. MMF. Improvement in immunosuppression by availability of drugs in the country and better diagnosis of infections have improved 1- and 5-year graft survival rates from 90.8% to 71.8% in 1994–1999 to 98.2% and 91% in the period 2014–2020 (Figure 5). The major cause of death in our patients is infection in >50% as compared to 33% in Europe and 45% in the region (Figure 6).

#### Donor Follow-Up

**FIGURE 7:**
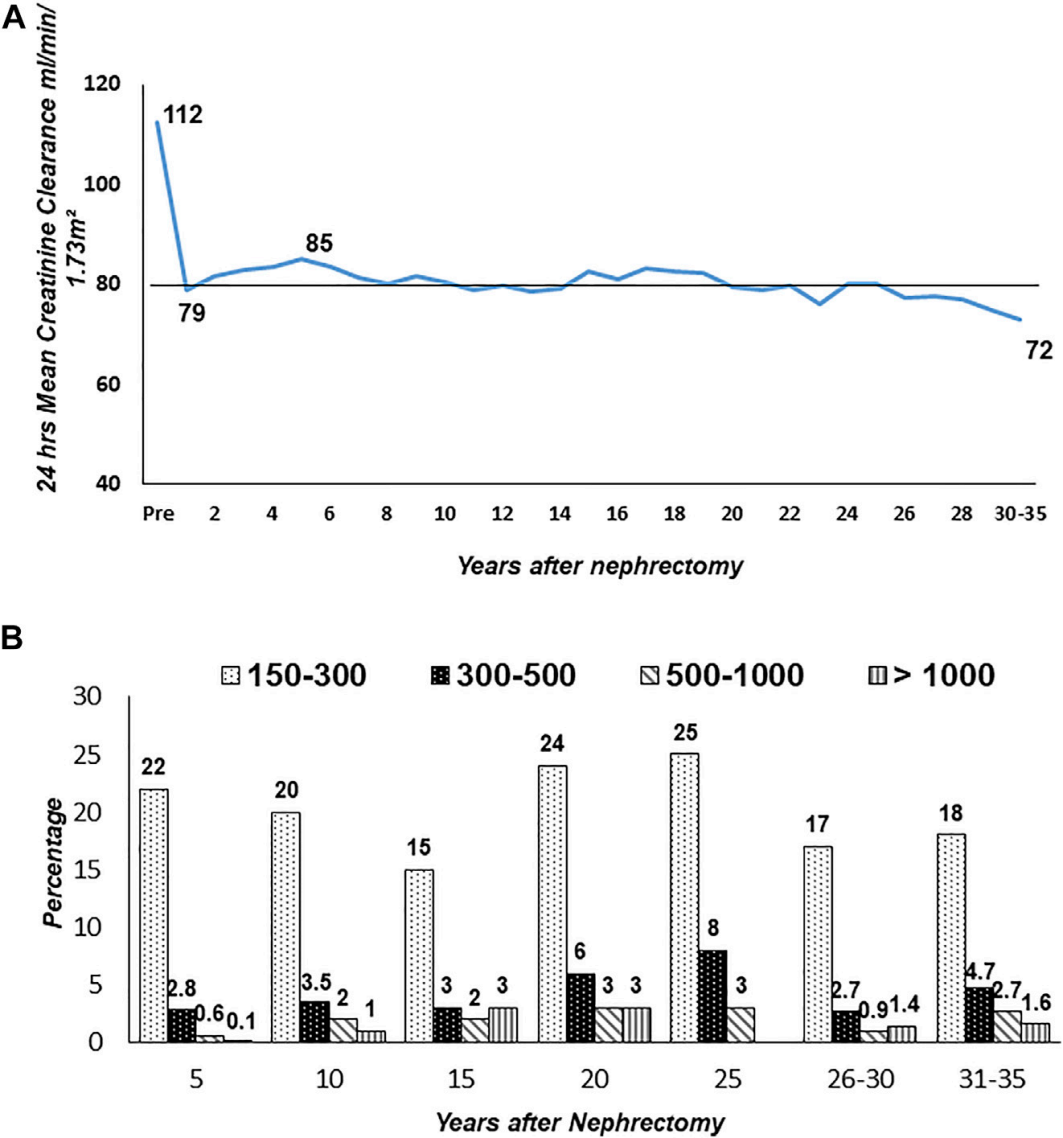
Long-term creatinine clearance and proteinuria in donors (*n* = 3,786). Figure **(A)**: Serial yearly creatinine clearance (CrCl). Figure **(B)**: Range of proteinuria (mg/24 h) by years after nephrectomy.

Donor clinic has registered 5,185 donors since its inception in 2000. Of the 5,185 registered, 4,883 (94%) are in follow-up. A total of 3,786 (77%) are in regular serial yearly follow-up. The rest have follow-up gaps of 2–5 years, especially >10–15 years after donation due to normal renal function and health. Mean post-donation age at >15 years was 49.5 ± 10.2 (Range 33–83), and 30–35 years was 62.0 ± 9.2 (Range 48–80). The mean serial CrCl in mL/min/1.73 m^2^ of 3,786 is donors given in Figure 7A. Pre-donation mean CrCl was 112 ± 23, which dropped to 79 ± 18 at 1 year. CrCl gradually increased to 85 ± 20 at 5 years and thereafter, there was an age-related fall to a mean of 72 ± 17 at 30–35 years. Overall protein excretion in mg/24 h at different time points is given in Figure 7B. The majority (76%) had protein excretion within the normal range <150 mg/24 h (76%) and 42 (1.1%) had protein >1,000 mg/24 h. In the follow-up period, 757 (20%) developed hypertension, 265 (7%) diabetes, and ESKD in 11 (0.29%). Overall 14 donors died, four in ESKD. The overall ESKD rate in donors was 2.5/10,000 person-years and mortality 4.5/10,000 person-years.

### Rehabilitation Program

Many of the recipients come from a low socio-economic background. The institute started a vocational training center where patients of all genders are given training in tailoring and computing, and beautician courses for women by qualified volunteers from the community. Furthermore, recipients and their donors are given employment on merit in the institute whenever possible. Presently over 175 are employed by the institute. Lastly, financial support is given to recipients to establish small businesses, e.g., home beauty parlor, tailor shop, vegetable, and fruit stalls.

## Discussion

Pakistan is an LMIC with paucity of RRT. The estimated incidence of ESKD is 100–150 pmp where the dialysis rate is 15 pmp and transplant 4.5 pmp. In view of this paucity, a model of community-government partnership was established where dialysis and transplantation were integrated and offered “Free of cost” to all who need it with lifelong follow-up of recipients and donors with medications. Daily, >1,500 patients are dialyzed and 6–8 transplants are performed per week. Overall, 1- and 5-year graft survival of 6,553 transplants are 97% and 87%. The funds contributed by the community and government to sustain all services in 2021 were $37.3 million.

### Access to Dialysis and Transplantation

#### Dialysis

In LMICs there are several problems associated with access to RRT. A major problem is that 20%–80% of the population resides in rural areas, while dialysis centers are located in urban centers [2, 3]. In our experience, although dialysis is free, patients have poor dialytic compliance due to long-distance and travel costs [13]. Establishment of satellite centers nearer to the doorstep of the patients have helped 1,875 patients access dialysis near their area of residence with substantial savings in time and travel costs. The other major issue is the cost of dialysis. In India where dialysis is available in rural settings, almost 50% stopped dialysis due to cost constrains [14]. In Nigeria, dialysis is available in public and private sector, however, patients cannot afford the costs resulting from a dialysis period of less than 1 month [15]. The reason for this drop out is high costs of dialysis in LMICs in the range of $13,510–$19,263 while free dialysis by public funding is only 22%–30%. When other funding models are included, e.g., out of pocket expenses, private funding, and models of public-private partnership the universal coverage for RRT is in the range 50%–70% in LMICs [1].

In a global survey based on World Bank income groups, public funding for chronic dialysis in LIC is 18%, and 22% in LMICs as compared to 58% in HIC [16]. In another study by International Society of Nephrology on dialysis funding in LMICs reported government contribution in 37.6%, out of pocket payment in 19.7%, employment insurance in 15.1% and private insurance 18.3% [17]. In summary, the majority of the patients are disfranchised from dialysis in LMICs due to lack of public services and high costs in the private sector. In fact, 50%–70% of the patients drop out of dialysis due to costs [18, 19]. In view of this, a number of LMICs have developed models of public private funding systems, similar to our model to offer dialysis to those who cannot due to cost constraints.

#### Transplantation

In LMICs the main treatment for ESKD is hemodialysis in a majority of the patients (range 10%–95%) and <1%–10% receive a kidney transplant (KT) [5]. A global survey of capacity for KT reported an incidence of 3.5 pmp for LIC and 4.3 pmp for LMICs [20]. The reason for this low activity is primarily absence of deceased donor programmes, thus transplants are only from living donors [2, 20]. In our own experience, the transplant rate is 1/3 of the dialysis rate and the main reason is the absence of deceased donors. Secondly, medical and social problems in potential donors, and thirdly patients with ESKD present late where pre-emptive transplants are not possible. Finally, the reason for low transplant rates is costs. The cost of KT in LMICs in the 1st year per person is $ 11,746 and after 1st year, it is $5,659/year [1]. These are beyond the means of the majority and when KT is available in an LIC it is publicly funded in 50%, and funded by a public-private partnership in 50%, while in an LMIC it is publicly funded in 27% and public-private in 54%.

When transplanted, the other issue is affording the cost of immunosuppression. In the majority, it is out of pocket or a public private partnership model. An international cross-sectional survey reported that funding for immunosuppression drugs was free at point of delivery in 20% in LICs and 42% in LMICs [21]. Therefore, the majority acquire drugs through out-of-pocket payment or other sources. The overall graft survival is therefore low in LMICs, 1- and 5-year range from 95%–83% and 93%–60% [2] as compared to HIC in Europe 98% at 1 year and 93.5% at 5 years. Providing free transplantation and life-long drugs, our overall graft survival rate at 1 year is 97% and 5 years 88%. Improving with time, the current survival rate is comparable to Europe 98.2% at 1 year and 91% at 5 years [9].

Paediatric transplant constitutes 4%–8% of the total living donor transplants in LMICs [22, 23]. The reasons for low activity in a report from Middle East countries identified delayed referrals, lack of infrastructure, and absence of dialysis facilities [23]. In a study from India, the main reasons were socioeconomic status, low wages, and distance from the transplant center [24]. Generally, in LMICs, lack of facilities and costs exclude children from transplantation. Transplant outcomes are also poor in LMICs with 1- and 5-year graft survival rates of 82%–98% and 44%–67% [25, 26] as compared to 99.5% and 97% in HIC (United States) [25]. Overcoming socioeconomic and logistic barriers in our Institute, children constitute 15% of all transplants with graft survival rates of 97% and 90% at 1 and 5 years.

### Cost Reduction Strategies for Increasing RRT

Costs of RRT are a burden for the government and patients in LMICs [27]. To reduce costs in dialysis one of the strategies employed is dialyzer reuse. This is not only cost-effective but also microbiologically safe [28]. In our experience, medium reuse was 7 days while others have reported average reuse of 3–10 times [18, 27]. In our experience, reuse allowed substantial savings by reducing the cost of dialysis to $0.64/dialysis from $4.5/dialysis. Reuse saved the institute $1.58 million in 2021. Early placement of AVF reduces costs of lines and costs of treatment of line associated infectious complications.

In transplantation, generic drugs provided a cost-effective option. The use of generic CyA and TAC at our institute for living donor transplants has shown comparable outcomes to living donor transplants in Europe. In fact, when newer generic drugs such as TAC and MMF have become available in the country, together with effective diagnosis and treatment of infections we have observed significant improvement in graft survival rates from 90.8% to 98.2%% at 1 year and 71.8%–91% at 5 years mostly using generic drugs. In a multicenter double-blinded randomized trial in Iran, generic CyA in comparison with a patent drug was found to be equally effective in terms of acute rejections, infections complications, and graft survival compared [29]. The same results were found in one-to-one conversion in stable renal transplant recipients [30]. In similar comparative studies of generic TAC vs. a patent drug, no difference was observed in rejection episodes, graft survival, and adverse events, e.g., infections and new onset diabetes [31, 32]. The use of generics thus offers LMICs a viable option as there are substantial savings, allowing more patients to be transplanted and given medications by public funding.

### Gender Disparities in RRT

A report by ERA-EDTA registry found that the lifetime risk of ESKD is 50% higher in men as compared to women [33]. In LMICs from Asia, ESKD rates in men ranged from 35%–65% [34]. Similarly, in a HIC (United States) the incidence of ESKD is 1.5 times higher in men than women [34]. A study from Pakistan reported that men constituted 51% of all CKD patients [35]. The disease is more prevalent in men, which is also confirmed by our own data where 60% of the patients on dialysis are men. Although disease is more prevalent in men, there appears to be a gender bias in dialysis due to cultural and logistic reasons.

Considering gender bias in transplantation, a study of 120 countries by Bikbov et al reported a Male:Female ratio of 10:2.5 for transplantation [36]. In LMICs there appears to be a gender bias where more men are recipients and more women are donors [37]. A meeting report from the Asia Pacific Region where data was based on National and Non-National resources showed that the proportion of female donors was 63%–78% in Bangladesh, 62%–65% in India, 53%–68% in Malaysia, 61% in Myanmar and 44% in Pakistan [37].

In contrast, there is predominance of male donors in some countries of the region. For instance, in Saudi Arabia 60%–70% are male donors [38]. The reason for this is the conservative Middle Eastern Society, which is culturally overprotective of women. A report from Iran based on data from the National Registry of 16,672 transplants showed that men constituted 62% of the recipients and 80% of the donors. Male predominance is likely due to economic, social, and cultural norms in Iran and perhaps their regulated compensation program may also attract male donors [39].

In a study from India, donation rates were compared from 2001–2009 and 2010–2018. There were improvements in male donor rates from 26.05% to 38.58% and male recipient rates decreased from 81.51% to 78.7% mainly due to awareness programs in the country [40].

In our experience men constitute 78% of the recipients and 54% of the donors. Although overall, women constituted 44% of the donors, however in paediatric transplants they constitute 58% and spousal transplants 85%. The majority of our patients belong to low a socio-economic class where men are the main financial earners and women homemakers. Women are socially and economically dependent on men and therefore easily volunteer to be donors. It may also be a social and cultural factor where women have a sense of obligation, love and altruism and care of the family [2, 11]. The main concern of both genders, especially men, is post donation health and wellbeing and the impact of donation on their ability to provide sustenance for their family. Our donor clinic has played an important role in bringing forward male donors. The donor clinic provides a forum for prospective donors to interact with kidney donors who have been in follow-up for more than 30 years. Good health of kidney donors gives confidence to prospective donors of a normal life post donation [11].

### The Way Forward for Dialysis and Transplantation in LMICs

In LMICs neither the government nor the patient has the capacity to pay for RRT. The governments can provide only 20%–30% of the patients free RRT and the patients *per capita* income is #3,000–24,000 per year while dialysis costs $13,510/year/person and transplant costs $11,746 in the first year. Several models of public-private partnership have been developed to fund RRT. Our model of community-government partnership has been sustained for over four decades with increasing support from the government and community. Dialysis at the doorstep of patients has increased accessibility and maintains equity in socio-economic and gender factors. This has given equal opportunity for women for dialysis and transplantation. Several models exist in LMICs where public-private partnerships have been able to provide RRT free of cost to patients. In India, the state government of Andhra Pradesh introduced an insurance scheme for poor households in 2007 called the Rajiv Aarogyasri Community Health Insurance Scheme (RACHIS), which offers free dialysis care [18]. In Guyana, a private-public partnership offers free transplantation with the help of a foundation called Subraj Foundation which has been sustained since 2007 [41]. In LMICs, models based on a government-private partnership offer a viable solution to enhance RRT.

## Conclusion

The results of our study where all RRT is provided “free of cost” to the disfranchised by a community government partnership may be duplicated in other LMICs. It may help overcome hurdles of logistics, economics, and gender and cultural biases inherent in LMICs.

## Data Availability

The raw data supporting the conclusions of this article will be made available by the authors, without undue reservation.

## Abbreviations

AKI, acute kidney injury; ATG, antithymocyte globulin; AVF, arterio-venous fistula; AZA, azathioprine; CrCl, creatinine clearance; CMV, cytomegalovirus; CTS, collaborative transplant study; CyA, cyclosporine; ESKD, end stage kidney disease; GDP, gross domestic product; HIC, high-income countries; HLA, human leucocyte antigen; IL-2, interleukin 2 antagonist; IMPDH, inosine-5′-monophosphate dehydrogenase; LIC, low-income countries; LMIC, low and low middle income countries; MMF, mycophenolate mofetil; PRA, panel reactive antibodies; RRT, renal replacement therapy; TAC, tacrolimus; TB, tuberculosis; UTI, urinary tract infection.

## References

[1] Yeung E Bello AK Levin A Lunney M Osman MA Ye F Current Status of Health Systems Financing and Oversight for End-Stage Kidney Disease Care: a Cross-Sectional Global Survey. BMJ Open (2021) 11(7):e047245. 10.1136/bmjopen-2020-047245 PMC8273453 34244267

[2] Rizvi SAH Zafar MN. Living Related Kidney Transplantation in Developing Countries: Life-Long Follow-Up of Recipients and Donors. In: Rohit A Abraham G, editors. Handbook of Renal Transplantation in Developing Countries. New Delhi, India: Oxford University Press (2020). p. 133–47.

[3] Nkunu V Wiebe N Bello A Campbell S Tannor E Varghese C Update on Existing Care Models for Chronic Kidney Disease in Low- and Middle-Income Countries: A Systematic Review. Can J Kidney Health Dis (2022) 9:20543581221077505. 10.1177/20543581221077505 35251672 PMC8894943

[4] Asian Development Bank. Key Indicators for Asia and the Pacific 2022. 53rd ed. Mandaluyong, Philippines: Asian Development Bank (2022). 10.22617/FLS220346-3

[5] Sahay M Jasuja S Tang SCW Alexander S Jha V Vachharajani T Aetiology, Practice Patterns and burden of End-Stage Kidney Disease in South Asia and South-East Asia: A Questionnaire-Based Survey. Nephrology (Carlton) (2021) 26(2):142–52. 10.1111/nep.13825 33169890 PMC7615902

[6] Divyaveer SS Ramachandran R Sahay M Singh Shah D Akhtar F Bello AK International Society of Nephrology Global Kidney Health Atlas: Structures, Organization, and Services for the Management of Kidney Failure in South Asia. Kidney Int Suppl (2021) 11(2):e97–e105. 10.1016/j.kisu.2021.01.006 PMC8084730 33981475

[7] Rizvi SA Naqvi SA Zafar MN Hussain Z Hashmi A Hussain M A Renal Transplantation Model for Developing Countries. Am J Transpl (2011) 11(11):2302–7. 10.1111/j.1600-6143.2011.03712.x 21883911

[8] Rizvi SA Naqvi SA Zafar MN Akhtar SF. A Kidney Transplantation Model in a Low-Resource Country: an Experience from Pakistan. Kidney Int Suppl (2013) 3(2):236–40. 10.1038/kisup.2013.22 PMC4089647 25018989

[9] Collaborative Transplant Study. Collaborative Transplant Study, Heidelberg University of Immunology (2023). Available from:https://www.ctstransplant.org (Accessed February 14, 2023).

[10] Zafar MN Wong G Aziz T Abbas K Rizvi SAH. Living Donor Risk Model for Predicting Kidney Allograft and Patient Survival in an Emerging Economy. Nephrology (Carlton) (2018) 23(3):279–86. 10.1111/nep.12983 27943514

[11] Ethics Committee of the Transplantation Society. The Consensus Statement of the Amsterdam Forum on the Care of the Live Kidney Donor. Transplantation (2004) 78(4):491–2. 10.1097/01.tp.0000136654.85459.1e 15446304

[12] Rizvi SA Zafar MN Jawad F Aziz T Hussain Z Hashmi A Long-term Safety of Living Kidney Donation in an Emerging Economy. Transplantation (2016) 100(6):1284–93. 10.1097/TP.0000000000001075 26854790

[13] Mazhar F Nizam N Fatima N Siraj S Rizvi SA. Problems Associated with Access to Renal Replacement Therapy: Experience of the Sindh Institute of Urology and Transplantation. Exp Clin Transpl (2017) 15(1):46–9. 10.6002/ect.mesot2016.O27 28260431

[14] Hemachandar R. Practice Pattern of Hemodialysis Among End-Stage Renal Disease Patients in Rural South India: A Single-center Experience. Saudi J Kidney Dis Transpl (2017) 28(5):1150–6. 10.4103/1319-2442.215134 28937077

[15] Ajayi S Raji Y Bello T Jinadu L Salako B. Unaffordability of Renal Replacement Therapy in Nigeria. Hong Kong J Nephrol (2016) 18:15–9. 10.1016/j.hkjn.2015.11.002

[16] Qarni B Osman MA Levin A Feehally J Harris D Jindal K Kidney Care in Low- and Middle-Income Countries. Clin Nephrol (2020) 93(1):21–30. 10.5414/CNP92S104 31397271

[17] Luyckx VA Smyth B Harris DCH Pecoits-Filho R. Dialysis Funding, Eligibility, Procurement, and Protocols in Low- and Middle-Income Settings: Results from the International Society of Nephrology Collection Survey. Kidney Int Suppl (2020) 10(1):e10–e18. 10.1016/j.kisu.2019.11.005 PMC7031691 32149005

[18] Shaikh M Woodward M John O Bassi A Jan S Sahay M Utilization, Costs, and Outcomes for Patients Receiving Publicly Funded Hemodialysis in India. Kidney Int (2018) 94(3):440–5. 10.1016/j.kint.2018.03.028 30143062

[19] Dhrolia MF Nasir K Imtiaz S Ahmad A. Dialyzer Reuse: Justified Cost Saving for South Asian Region. J Coll Physicians Surg Pak (2014) 24(8):591–6. 25149841

[20] Mudiayi D Shojai S Okpechi I Christie EA Wen K Kamaleldin M Global Estimates of Capacity for Kidney Transplantation in World Countries and Regions. Transplantation (2022) 106(6):1113–22. 10.1097/TP.0000000000003943 34495014 PMC9128615

[21] Bello AK Levin A Lunney M Osman MA Ye F Ashuntantang GE Status of Care for End Stage Kidney Disease in Countries and Regions Worldwide: International Cross Sectional Survey. BMJ (2019) 367:l5873. 10.1136/bmj.l5873 31672760

[22] Rizvi SA Sultan S Zafar MN Naqvi SAA Lanewala AA Hashmi S Pediatric Kidney Transplantation in the Developing World: Challenges and Solutions. Am J Transpl (2013) 13(9):2441–9. 10.1111/ajt.12356 23865679

[23] Saeed B. Pediatric Kidney Transplantation in the Middle East: Challenges and Solutions. Exp Clin Transpl (2022) 20(3):7–14. 10.6002/ect.PediatricSymp2022.L2 35570592

[24] Pais P Blydt-Hansen TD Michael Raj JA Dello Strologo L Iyengar A. Low Renal Transplantation Rates in Children with End-Stage Kidney Disease: A Study of Barriers in a Low-Resource Setting. Pediatr Transpl (2021) 25(2):e13867. 10.1111/petr.13867 33058452

[25] Chua A Cramer C Moudgil A Martz K Smith J Blydt-Hansen T Kidney Transplant Practice Patterns and Outcome Benchmarks over 30 Years: The 2018 Report of the NAPRTCS. Pediatr Transpl (2019) 23(8):e13597. 10.1111/petr.13597 31657095

[26] Iyengar A McCulloch MI. Paediatric Kidney Transplantation in Under-resourced Regions-A Panoramic View. Pediatr Nephrol (2022) 37(4):745–55. 10.1007/s00467-021-05070-3 33837847 PMC8035609

[27] Alexander S Jasuja S Gallieni M Sahay M Rana DS Jha V Impact of National Economy and Policies on End-Stage Kidney Care in South Asia and Southeast Asia. Int J Nephrol (2021) 2021:6665901. 10.1155/2021/6665901 34035962 PMC8118744

[28] Ribeiro IC Roza NAV Duarte DA Guadagnini D Elias RM Oliveira RB. Clinical and Microbiological Effects of Dialyzers Reuse in Hemodialysis Patients. J Bras Nefrol (2019) 41(3):384–92. 10.1590/2175-8239-JBN-2018-0151 30720850 PMC6788851

[29] Khatami SM Taheri S Azmandian J Sagheb MM Nazemian F Razeghi E One-Year Multicenter Double-Blind Randomized Clinical Trial on the Efficacy and Safety of Generic Cyclosporine (Iminoral) in De Novo Kidney Transplant Recipients. Exp Clin Transpl (2015) 13(3):233–8. 10.6002/ect.2013.0139 26086833

[30] Cortinovis M Gotti E Trillini M Carrara F Gaspari F Ruggenenti P Conversion from Brand-Name Neoral to the Generic Ciqorin in Stable Renal Transplant Recipients. Nephron (2017) 135(3):173–80. 10.1159/000453671 27941326

[31] Melilli E Crespo E Sandoval D Manonelles A Sala N Mast R De Novo use of a Generic Formulation of Tacrolimus versus Reference Tacrolimus in Kidney Transplantation: Evaluation of the Clinical Results, Histology in Protocol Biopsies, and Immunological Monitoring. Transpl Int (2015) 28(11):1283–90. 10.1111/tri.12626 26088437

[32] Son SY Jang HR Lee JE Yoo H Kim K Park JB Comparison of the Long-Term Efficacy and Safety of Generic Tacrobell with Original Tacrolimus (Prograf) in Kidney Transplant Recipients. Drug Des Devel Ther (2017) 11:203–10. 10.2147/DDDT.S118154 PMC5238812 28138224

[33] ERA-EDTA. ERA-EDTA Registry Annual Report 2017 (2017). Available from:http://www.era-online.org/wp-content/uploads/2022/11/ERA-Registry-Annual-Report-2017.pdf (Accessed February 14, 2023).

[34] Ricardo AC Yang W Sha D Appel LJ Chen J Krousel-Wood M Sex-related Disparities in CKD Progression. J Am Soc Nephrol (2019) 30(1):137–46. 10.1681/ASN.2018030296 30510134 PMC6317604

[35] Salman B Imtiaz S Qureshi R Dhrolia MF Ahmad A. The Causes of Chronic Kidney Disease in Adults in a Developing Country. J Nephrol Ren Dis (2017) 1:1. 10.4172/2576-3962.1000105

[36] Bikbov B Perico N Remuzzi G. On Behalf of the GBD Genitourinary Diseases Expert Group Disparities in Chronic Kidney Disease Prevalence Among Males and Females in 195 Countries: Analysis of the Global Burden of Disease 2016 Study. Nephron (2018) 139(4):313–8. 10.1159/000489897 29791905

[37] Kim Y Ahmed E Ascher N Danguilan R Hooi LS Hustrini NM Meeting Report: First State of the Art Meeting on Gender Disparity in Kidney Transplantation in the Asia-Pacific. Transplantation (2021) 105(9):1888–91. 10.1097/TP.0000000000003841 34416749

[38] Guella A Mohamed E. Donor and Recipient Gender Distribution in a Saudi Kidney Transplant Center. Transpl Proc (2011) 43(2):415–7. 10.1016/j.transproceed.2011.01.049 21440721

[39] Taheri S Alavian SM Einollahi B Nafar M. Gender Bias in Iranian Living Kidney Transplantation Program: a National Report. Clin Transpl (2010) 24(4):528–34. 10.1111/j.1399-0012.2009.01120.x 19843109

[40] Bhuwania S Saxena S Bansal R Goel R. Gender Bias in Kidney Donation in India: Has it Changed Over the Past 2 Decades? Transpl Proc (2020) 52(6):1665–70. 10.1016/j.transproceed.2019.12.056 32417037

[41] Guy-Frank CJ Persaud K Butsenko D Jindal RM Guy SR. Developing a Sustainable Renal Transplant Program in Low- and Middle-Income Countries: Outcome, Challenges, and Solutions. World J Surg (2019) 43(11):2658–65. 10.1007/s00268-019-05093-w 31363826

